# Correct folding of an α-helix and a β-hairpin using a polarized 2D torsional potential

**DOI:** 10.1038/srep10359

**Published:** 2015-06-03

**Authors:** Ya Gao, Yongxiu Li, Lirong Mou, Bingbing Lin, John Z. H. Zhang, Ye Mei

**Affiliations:** 1 State Key Laboratory of Precision Spectroscopy, Department of Physics and Institute of Theoretical and Computational Science, East China Normal University, Shanghai 200062 China; 2 Institutes for Advanced Interdisciplinary Research, East China Normal University, Shanghai 200062 China; 3 NYU-ECNU Center for Computational Chemistry at NYU Shanghai, Shanghai China 200062

## Abstract

A new modification to the AMBER force field that incorporates the coupled two-dimensional main chain torsion energy has been evaluated for the balanced representation of secondary structures. In this modified AMBER force field (AMBER03^2D^), the main chain torsion energy is represented by 2-dimensional Fourier expansions with parameters fitted to the potential energy surface generated by high-level quantum mechanical calculations of small peptides in solution. Molecular dynamics simulations are performed to study the folding of two model peptides adopting either α-helix or β-hairpin structures. Both peptides are successfully folded into their native structures using an AMBER03^2D^ force field with the implementation of a polarization scheme (AMBER03^2D^p). For comparison, simulations using a standard AMBER03 force field with and without polarization, as well as AMBER03^2D^ without polarization, fail to fold both peptides successfully. The correction to secondary structure propensity in the AMBER03 force field and the polarization effect are critical to folding Trpzip2; without these factors, a helical structure is obtained. This study strongly suggests that this new force field is capable of providing a more balanced preference for helical and extended conformations. The electrostatic polarization effect is shown to be indispensable to the growth of secondary structures.

Unraveling the mechanism of protein folding has been a grand challenge in biological science for decades^1^,^2^,^3^. Relentless experimental effort has been devoted to the study of protein folding and unfolding under various conditions^4^,^5^,^6^. In parallel, theoretical and computational methods are playing an increasingly important role in this field. With continuous advances in computer technology, the lengthy time scales required for protein folding by computer simulation have lessened significantly. For example, a recent atomistic simulation of a 58-residue protein BPTI conducted by Shaw *et al.* achieved results in one millisecond^7^. However, the successful application of computer simulations is impeded by the accuracy of the force fields employed. Defects in potential energy can strongly bias the simulation result toward incorrect conformations^8^,^9^.

Despite cases of excellent agreement between experiments and computer simulations reported in the literature^10^,^11^,^12^, some inherent deficiencies of conventional force fields are well known. For example, the *α*-helical propensity of the AMBER03^13^ force field is too high relative to experimental measurements, while that of AMBER99SB^14^ is arguably too low^15^. CHARMM27 also has a helical propensity. A 10-μs simulation of the all *β*-structure of the Pin WW domain starting from an unfolded conformation resulted in a purely helical structure when run with CHARMM27^9^. Thus, developing a well-balanced force field for various secondary structures poses a challenge to computational biology. Best *et al.* calibrated the backbone rotation terms in the widely used AMBER03, AMBER99SB, and CHARMM22/CMAP force fields, which resulted in a significant improvement in reproducing experimental residual dipolar couplings^16^,^17^. Lindorff *et al.* refined the χ_1_ torsion potentials for amino acid side chains in AMBER99SB and found much closer agreement between the simulations and NMR experiments for the rotameric states of all four modified residues^18^. Other torsional corrections to CHARMM, GROMOS, and OPLS force fields have also been suggested^19^,^20^,^21^,^22^,^23^. Lindorff *et al.* performed a systematic validation of these refined force fields against experimental data, and suggested that force fields have improved over time. Nevertheless, they also highlighted residual deficiencies in force fields and noted areas for future improvement, such as temperature dependence^24^. Along with defects in torsional potential, the lack of an inhomogeneous polarization effect is also a likely reason for the failure of some protein folding simulations. The polarization effect has been shown to be critical to the success of protein folding^25^,^26^,^27^,^28^,^29^,^30^,^31^,^32^,^33^. The crucial role played by the polarization effect in protein self-assembly has also been observed^34^,^35^.

In widely used AMBER force fields, the main chain torsion terms are treated individually, for φ (C-N-Cα-C) and ψ (N-Cα-C-N), as one-dimensional Fourier series. Due to the limited number of parameters for the torsion term, parameterization mainly focuses on a small portion of space near some energy basins. This leads to errors in torsion energy for inter-basin regions and barriers^36^,^37^,^38^. As these two main chain torsion energies are not physically separable in the potential energy map, a more desirable choice is to expand the torsion energy function by using orthogonal basis sets of both φ and ψ in a coupled fashion. Recently, we calibrated the AMBER03 and AMBER99SB force fields by replacing the one-dimensional Fourier series for φ and ψ with a coupled 2-dimensional (2D) main chain torsion energy (denoted AMBER03^2D^ and AMBER99SB^2D^)^39^. These two force fields are more balanced among various conformations than the original AMBER force fields. This idea has also been adopted by Lwin and Luo in their ff99ci force field^40^. However, because the model system used for parameterization is an alanine dipeptide, the electrostatic polarization effect along main chain hydrogen bonds has not been included, which is essential when ordered secondary structures such as α-helices and β-strands are formed. Therefore, the performance of AMBER03^2D^ and AMBER99SB^2D^ in molecular dynamics simulations of real proteins still remains to be examined.

In our previous work, we have assessed the performance of these force fields by checking the simulated conformation distribution and NMR J coupling of short alanine peptides. In this work, we evaluate the reliability of AMBER03^2D^ in the folding simulations of two short peptides representing, respectively, an α-helix and a β-sheet. Our results show that when combining this new force field and the electrostatic polarization effect, both peptides can fold into their native structures. For comparison, the AMBER03 force field is too helical to fold the β-hairpin peptide whether the polarization effect is included or excluded.

## Methodology

### 2D torsion force field

A detailed explanation of the implementation and parameterization of this coupled 2D torsion potential can be found in our previous work^39^. Here we give a brief introduction. The alanine dipeptide (AD) was chosen as a model system for parameterization. The fitting of main chain torsion energy at a molecular mechanical (MM) level aims to reproduce the total energy of AD at a quantum mechanical (QM) level. Energy calculations are conducted on 24 × 24 grid points in the 2-dimensional space of main chain torsions with a 15° interval. All quantum mechanical calculations are performed at the M06 2X/aug-cc-pvtz//HF/6-31G** level by Gaussian 09^41^,^42^. At the QM level, the solvation effect is implemented by the integral equation formalism variant of the polarizable continuum model (IEFPCM)^43^ in both optimization and single point calculations. The QM potential energy of AD can be written as


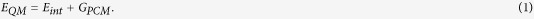


The potential energy of AD at the MM level is expressed as


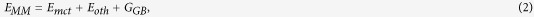


in which 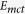 and 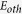 are the main chain torsion related energy terms and other internal contributions, respectively, and 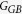 is the generalized Born solvation free energy. By equalizing the MM and QM energies, the main chain torsion term can be calculated as


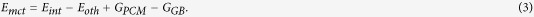


In the AMBER force field, the torsion energy is expressed as a series of separate Fourier expansions for φ and ψ. However, a more rational method involves decomposing the main chain torsion energy map with a double Fourier series, after which the expansion coefficients can be shown in matrix form as


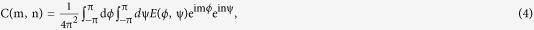


in which 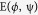 are the main chain torsion potential energies (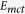). At any value of 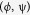, the potential energy and the force acting on atom *k* involved in backbone torsions can be calculated, respectively, as


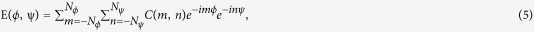


and


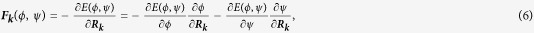


where ***R***_***k***_ is the position of atom *k*.

### Molecular dynamics simulations for benchmark systems

In our previous study, the accuracy of 2D force fields was validated with an implicit water model, showing close agreement with experimental results. In this work, an explicit water model (TIP3P) was used. We chose some well-studied systems for validations, for which experimental J coupling data are available. The benchmark systems used include capped dipeptides (Ace-X-NME, X
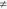
P), tripeptides (XXX, X
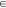
{A, G, V}; GYG, Y
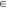
{A, V, F, L, S, E, K, M}), and alanine tetrapeptide. All systems used have available NMR data in the form of chemical shifts, J couplings, or both. Secondary structures for dipeptides are classified into “
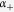
” (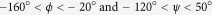), “
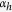
” with a more stringent definition (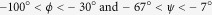), “
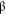
” (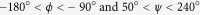 or 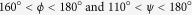), and “PPII” (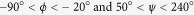) as used by Best *et al.*, and compared with experimental values from vibrational spectroscopy^44^. Along with the secondary structure population, 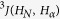 coupling was calculated from the Karplus equation to inspect the intrinsic conformational distributions of different force fields. The parameters used in this work are taken from Hu *et al.*^45^, which is an experimental investigation of ubiquitin averaged over various residues.


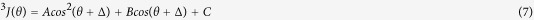


### Electrostatic polarization effect

In classic molecular mechanics, main chain hydrogen bonds stabilize the secondary structure through Coulomb interactions. However, when a hydrogen bond forms or breaks, the electron density redistributes to lower the total energy of the system. Therefore, electrostatic polarization plays an important role in protein folding. We employ a recently developed polarization model^31^. This model is based on the theoretical study of two alanine dipeptides that interact through a main chain hydrogen bond. By alternating the distance between the donor and the acceptor (d) from 2.5 Å to 6.5 Å and fixing the other degrees of freedom (DoF), a series of quantum mechanical calculations can be performed to obtain the electronic structures of the interacting dipeptide pair. In the calculation of each dipeptide, the other dipeptide is taken as a group of background charges located at the nuclei to polarize the electronic structure of the dipeptide studied. Electrostatic potentials on grids around the residue are calculated based on polarized wave functions, and new atomic charges are obtained using the restrained electrostatic potential (RESP) charge fitting method^46^. Only the charge transfers between the N and H atoms and between the C and O atoms involved in the hydrogen bond are allowed. Other atoms have their atomic charges fixed to the original AMBER charges. Quantum mechanical calculations and charge fitting are performed iteratively until convergence is reached. Finally, the charge alternations of N and O atoms can be fitted to single exponential functions of d as


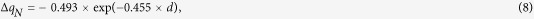


and


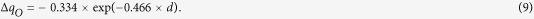


This polarization model has proven to be quite effective at improving the thermodynamics of 2I9M^31^ and the much longer helix 2KHK^32^.

### Folding simulations of α-helix and β-hairpin proteins

2I9M is a “*de novo*” designed 17-residue helical peptide. Its native structure is determined by NMR experimentation at 283K and pH = 5.0. Trpzip2 is a 12-residue mini-protein (PDB ID: 1LE1) designed by Cochran *et al.*^47^ This peptide adopts a β-hairpin structure enabling π-stacking between tryptophan residues. The salt bridge between GLU5 and LYS8 may play a role in stabilizing the turn region of this protein^48^. In this paper, we employ both replica exchange molecular dynamics (REMD) and direct molecular dynamics simulations to study these two peptides. In REMD simulations, high temperature replicas can easily surmount the energy barrier and explore the phase space more efficiently than standard molecular dynamics at room temperature^49^. Free energy is calculated by the Weighted Histogram Analysis Method (WHAM)^50^,^51^ as


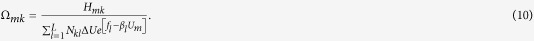


Direct molecular dynamics simulations are performed at room temperature. More simulation details can be found in the section **Simulation Details**. The force fields employed include AMBER03, AMBER03 plus polarization (denoted as AMBER03p), AMBER03^2D^, and AMBER03^2D^ plus polarization (denoted as AMBER03^2D^p).

## Results and Discussion

### Accuracy examination of AMBER^2D^ force fields for benchmark systems in an explicit water model

**Figure 1:**
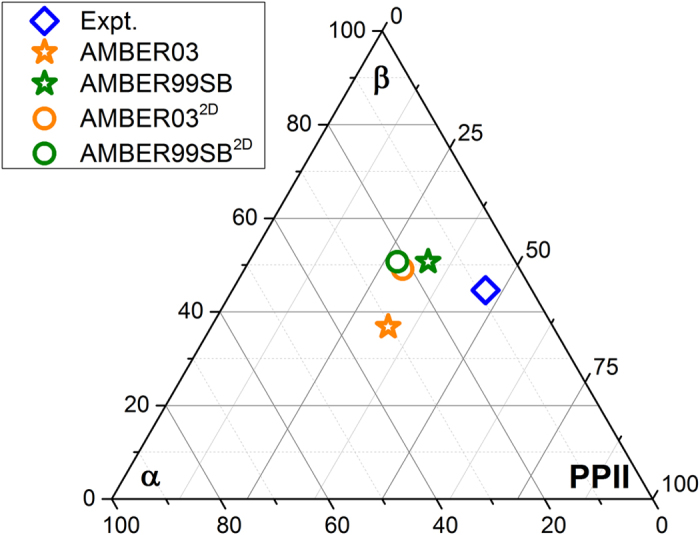
The simulated average conformational population for 19 dipeptides under AMBER03, AMBER03^2D^, AMBER99SB, and AMBER99SB^2D^ force fields. Each axis denotes the population of 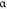, 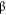, and PPII from the bottom, clockwise. The corners of the triangle represent distributions with 100% 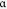, 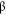, or PPII, respectively.

The torsional parameters used are derived from an alanine dipeptide. Thus, a key question is whether the performance of these 2D force fields is consistent among different amino acids. First, we evaluate the quality of AMBER03^2D^ and AMBER99SB^2D^ force fields for 19 amino acids using molecular simulations and compare these results with those of AMBER03 and AMBER99SB force fields. The torsional parameters for Proline are left intact as in the original AMBER force field. The conformational populations from simulations using the AMBER03, AMBER03^2D^, AMBER99SB, and AMBER99SB^2D^ force fields are shown in Fig. 1. Because of the uncertainties in structure identification and the fitting procedure used in experimental analysis, this comparison is less direct than comparisons of NMR couplings. Despite this limitation, population analysis provides several insights. The 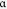 population in dipeptide simulations under the AMBER03 force field is nearly one fold larger than that under the AMBER99SB force field. After the 2D torsional potential correction, the secondary structural populations for AMBER03^2D^ and AMBER99SB^2D^ force fields are nearly the same, which indicates the well-balanced character of these 2D force fields. There is still a large discrepancy between calculations and experimental results, which may be due to systematic error in the experiment.

**Figure 2:**
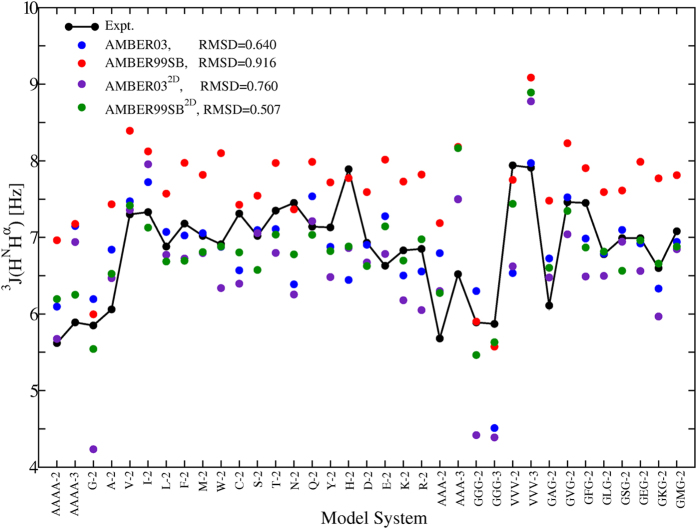
The simulated 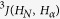 under AMBER03, AMBER03^2D^, AMBER99SB, and AMBER99SB^2D^ force fields, and the RMSDs from experimental measurements.

Along with these 19 dipeptides, we selected other benchmark systems for validation. Following Pande *et al.*^52^, we calculated the 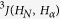, which indicates the intrinsic secondary distribution of force fields. The result is shown in Fig. 2 for various systems. From this figure we can see that the torsional parameters derived from single amino acids are not readily applicable to all residue types. Large deviations can be observed for specific residues. The difference in solvent models between QM and MM calculations is also a possible source error in the revised force fields. For the AMBER03^2D^ force field, glycine is the largest contributor of error, which is consistent with previous observations^53^. However, the AMBER99SB^2D^ force field performs significantly better than its predecessor, leading to noticeable improvements in force field accuracy.

### REMD simulations with the AMBER03^2D^ force field

**Figure 3:**
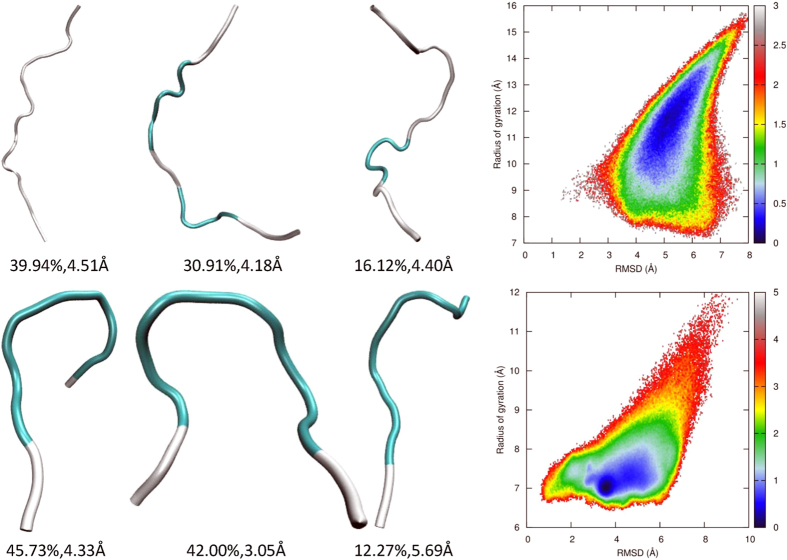
Representative structures from clustering analysis and the free energy landscape in the space of RMSD from the native structure and the radius of gyration for 2I9 M (top panel) and trpzip2 (bottom panel) using the AMBER03^2D^ force field at 300 K, from the REMD simulations.

Nucleation and helical growth are favored by the formation of main chain hydrogen bonds but are retarded by the desolvation of polar atoms and the loss of conformational entropy. The model peptide primarily adopts extended conformations, including β and PPII, under the AMBER03^2D^ force field^39^. This intrinsic conformational propensity of the AMBER03^2D^ force field results directly from the quantum mechanical potential energy surface of the alanine dipeptide and agrees with experimental observations^44^,^54^,^55^. The folding simulation of 2I9M can verify whether the enthalpic gain in the formation of a helix overcomes hindrances and shifts the population from extended conformations to helical structures. Conformation clustering analysis shows that there is no nucleus formed in the major clusters shown in Fig. 3. The free energy landscape shows that 2I9M covers a broad area in the space of RMSD and Rg under this force field, illustrating that there is no strongly preferential conformation adopted by this peptide. The minimal free energy structure has a much larger Rg than that of the native structure. The failure of peptide 2I9M to fold indicates that hydrogen bonding is not strong enough to compete with desolvation and the loss of entropy.

Clustering analysis of the AMBER03^2D^ trajectory shows that the peptide trpzip2 has a large portion of its population (42%) in conformations with a turn structure, as shown in Fig. 3. These conformations are close to the native structure, but further advancement to the folded state by the formation of main chain hydrogen bonds and π-stacking of tryptophan side chains is not achieved. There is only one minimum in the free energy landscape mapped to RMSD and Rg. Although located in the unfolded domain, the compactness of the conformations in this minimum is very close to the native structure. The free energy of the native structure is approximately 2 kcal/mol higher than that of this minimum. Therefore, there is a potential energy penalty in dewetting the backbone polar atoms, and entropy loss impedes the folding of trpzip2.

### REMD simulations with the AMBER03^2D^p force field

**Figure 4:**
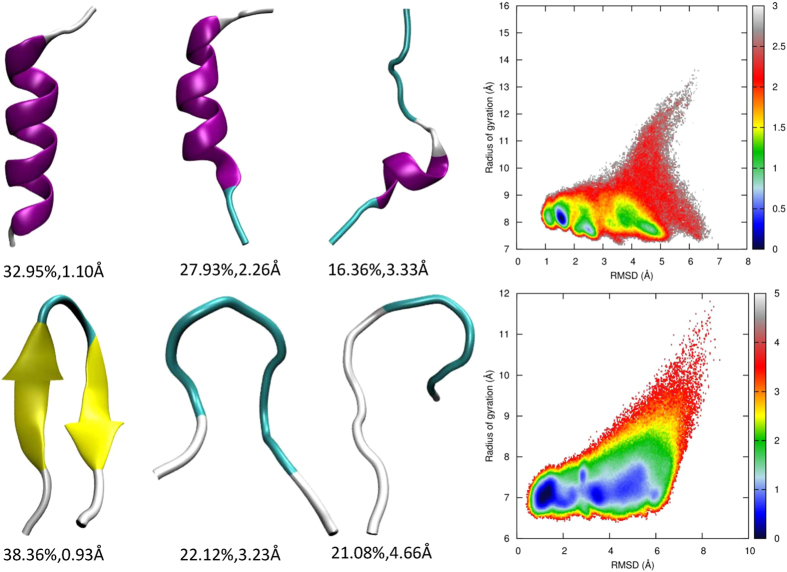
Representative structures from clustering analysis and the free energy landscape in the space of RMSD from the native structure and the radius of gyration for 2I9 M (top panel) and trpzip2 (bottom panel) using the AMBER03^2D^p force field at 300 K, from the REMD simulations.

During the parameterization of the AMBER03^2D^ force field, quantum mechanical (QM) calculations are performed for various conformations of the alanine dipeptide. The main chain torsion term is fitted by minimizing the difference between the QM and MM potential energies. However, this model system is too small to form main chain hydrogen bonds as in typical helices or β-strands. Therefore, the electrostatic polarization effect due to hydrogen bond formation cannot be captured in the AMBER03^2D^ force field. The pairwise AMBER charge is based on a mean-field approximation. The heterogeneous polarization in a hydrogen bonded residue pair is not explicitly included. When hydrogen bonds occur, the electron density redistributes, giving rise to enhanced attraction between the donor and the acceptor. Therefore, it is necessary to incorporate a polarization effect into the AMBER03^2D^ force field. The polarization effect over the AMBER03^2D^ force field shifts the population of 2I9M significantly toward the native conformation. The RMSD distribution peaks at approximately 1.7 Å, which is conventionally regarded as the folded region. Nonetheless, there is still a large population residing in the unfolded region. As shown in Fig. 4, the largest cluster contains 32.95% of conformations with the centroid structure a fully folded helix. The second largest cluster contains 27.93% of the conformations, of which the centroid structure is partially unfolded. The peptide covers a narrow phase space with the global minimum in the folded domain and two local minima in the unfolded domain. These two local minima adopt even more compact conformations (with smaller Rg) than the native structure, corresponding to two partially folded structures. One has two nascent helix fragments in the middle of the chain, and the other has a longer helix segment with 2.5 consecutive turns (data not shown). The specific heat 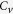 can be calculated from the thermal fluctuation of total energy, which peaks at the melting temperature. Under the AMBER03^2D^p force field, the simulated 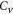 of 2I9M is 315 K.

As the AMBER03^2D^ force field can generate a considerable population of conformations with a turn structure, a polarization effect can help these structures achieve a native fold by strengthening the main chain hydrogen bonds in trpzip2. As expected, clustering analysis shows that the dominant cluster is a reservoir of folded structures, as shown in Fig. 4, which cover over 38% of conformations. The RMSD of the centroid structure is below 1 Å. Other clusters are archives of unfolded structures. A large populations of unfolded structures indicates that the native structure is not the exclusively sampled conformation for trpzip2 in aqueous solution at 300 K. The global free energy minimum conformation under AMBER03^2D^ is now only a local minimum. The global minimum under the AMBER03^2D^p force field is located in the folded region. These two minima are separated by a low barrier of approximately 1 kcal/mol.

**Figure 5:**
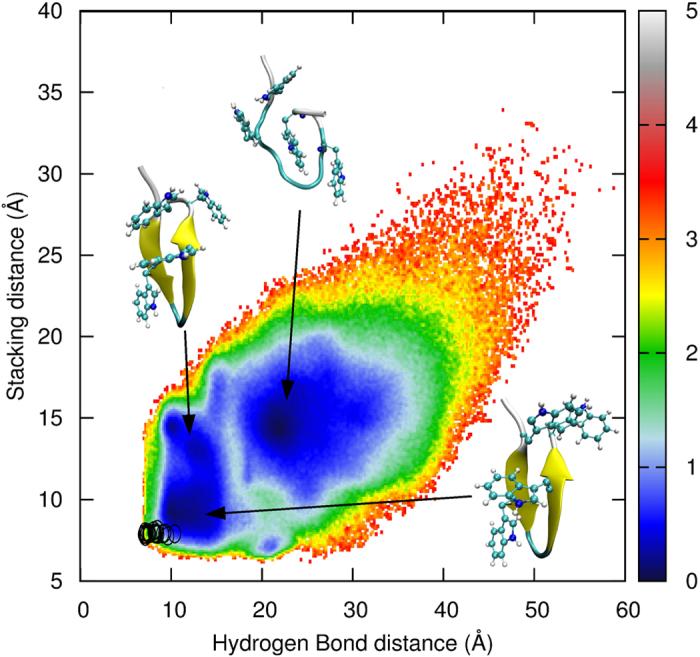
Free energy landscape of trpzip2 in the space of the length of inter-strand main chain hydrogen bonds (d) and the distance between stacking tryptophan side chains (l) at 300 K from the REMD simulation using the AMBER03^2D^p force field. The locations of the experimental structures in the PDB file are shown as black circles.

The folding pathway of trpzip2 is still an ongoing debate^40^,^56^,^57^,^58^,^59^,^60^,^61^,^62^,^63^,^64^,^65^, because the simulated pathway relies on the force field that is employed. Whether the formation of inter-strand hydrogen bonds occurs prior to the formation of π-stacking is still unknown. We map the free energy landscape into the hydrogen bond distance *d* and the stacking distance *l* (see the section **Simulation Details** for definitions) as shown in Fig. 5. The experimental structures (shown as black circles) are very close to the global minimum of the free energy landscape. The unfolding of trpzip2 begins with the separation of the π-stacked tryptophan side chains. As can be read from the free energy landscape, this intermediate state has a longer stacking distance but a similar hydrogen bond distance to the native structure. The next step of unfolding is the breaking of the main chain hydrogen bonds of trpzip2, during which it crosses a free energy barrier and enters the unfolded region. In the unfolded region, trpzip2 can adopt certain conformations with formed π-stacking but without the native main chain hydrogen bonds. However, if beginning from these π-stacking conformations, trpzip2 must surmount a barrier higher than that mentioned above before entering the folded region. Therefore, our REMD simulation employing the AMBER03^2D^p force field supports the hydrogen bond-first mechanism of trpzip2 folding. The simulated 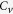 under the AMBER03^2D^p force field is 335 K, which is close to the experimentally observed value of 345 K^47^. This 10 K difference can be explained by the underestimated viscous drag in the GB solvation model.

### Comparative study of the AMBER03 and AMBER03p force fields

**Figure 6:**
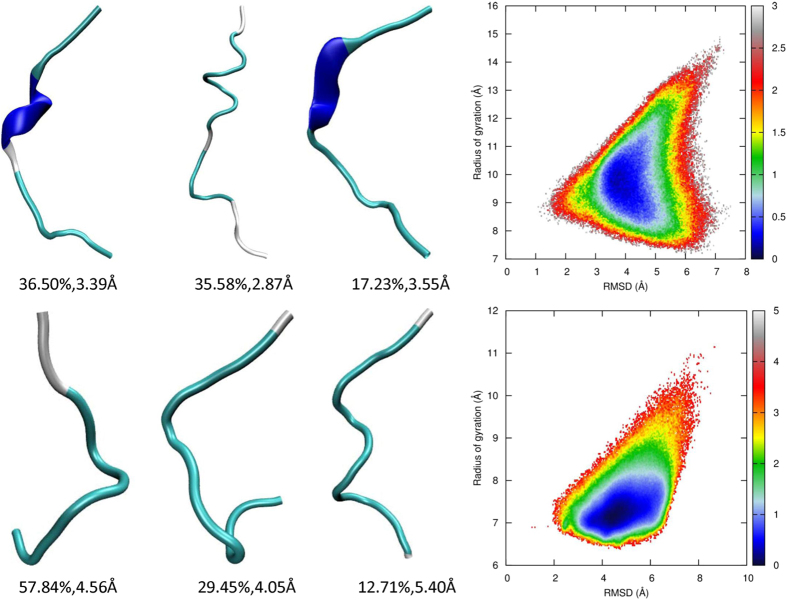
Representative structures from clustering analysis and the free energy landscape in the space of RMSD from the native structure and the radius of gyration for 2I9 M (top panel) and trpzip2 (bottom panel) using the AMBER03 force field at 300 K, from the REMD simulations.

**Figure 7:**
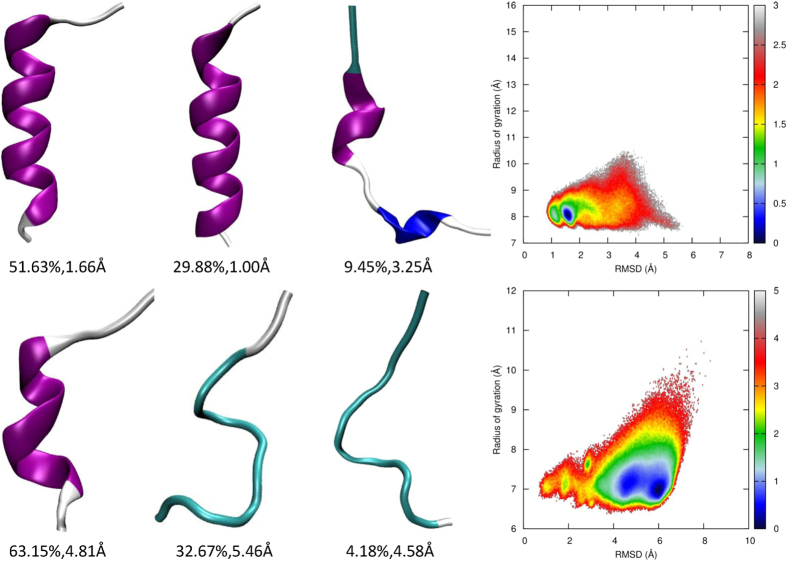
Representative structures from clustering analysis and the free energy landscape in the space of RMSD from the native structure and the radius of gyration for 2I9 M (top panel) and trpzip2 (bottom panel) using the AMBER03p force field at 300 K, from the REMD simulations.

The AMBER03 force field is biased toward helical conformations. It might not be difficult for protein 2I9M to fold under the AMBER03 force field. However, in our previous study^31^, the simulated melting temperature of 2I9M under AMBER03 and the GB^OBC^ solvation model^66^ was much lower than the experimentally measured value, despite the GB^OBC^ solvation model also being biased toward helical conformations^67^. In this study, we employ the GB^MSMCO^ solvation model^68^, which is more balanced among conformations. As expected, peptide 2I9M cannot fold to its native state under the AMBER03 force field. The root mean square deviation (RMSD) from the native structure fluctuates between 2 and 7 Å. Clustering analysis of the folding trajectory at 300 K shows that, in the majority of trajectories, the peptide has only a helical nucleus and most of its residues are in nonstructured coil conformations, as shown in Fig. 6. This indicates that hydrogen bonds are not strong enough to compensate for the desolvation penalty and entropy loss during the growth of the helix. The conformations achieved are distributed very broadly along Rg and RMSD ranges. The free energy minimum has a less compact structure with a larger Rg (9.5 Å) than those of the native structures (Rg ≈ 8.57 Å). While employing the AMBER03p force field, the first two dominant conformation clusters are both reservoirs of folded structures that sum to over 80% of the achieved conformations, as shown in Fig. 7, which illustrates that polarization effectively shifts the conformation distribution of this peptide to its native structure. The free energy landscape shows that peptide 2I9M covers a much narrower phase space under AMBER03p than it does under the pairwise AMBER03 force field. The Rg of the minimal free energy conformation is very close that of the native structure. Therefore, the AMBER03p force field is very effective at folding this short helical peptide, and the electrostatic polarization effect plays a critical role in maintaining the secondary structure.

As the AMBER03 force field is biased toward helical conformations, the simulated folding of a protein in a β conformation is a challenge to this force field. Conformation clustering analysis, which shows that trpzip2 adopts mainly nonstructured conformations, clearly indicates that the native conformation of trpzip2 is not the preferred conformation under the AMBER03 force field at 300 K, as shown in Fig. 6. Turning on the polarization effect does not improve the result. Indeed, conformations with a short helical fragment appear as observed in Fig. 7. This failure is consistent with the previous observations that AMBER03 is biased toward helical conformations^15^, and that the polarization effect further stabilizes misfolded structures. There is only one minimum in the free energy landscape under the AMBER03 potential, as shown in Fig. 6. Polarization splits this free energy well into two not-well-separated minimums that are still located in the unfolded zone (see Fig. 7).

### Direct MD simulation of 2I9M and trpzip2

**Figure 8:**
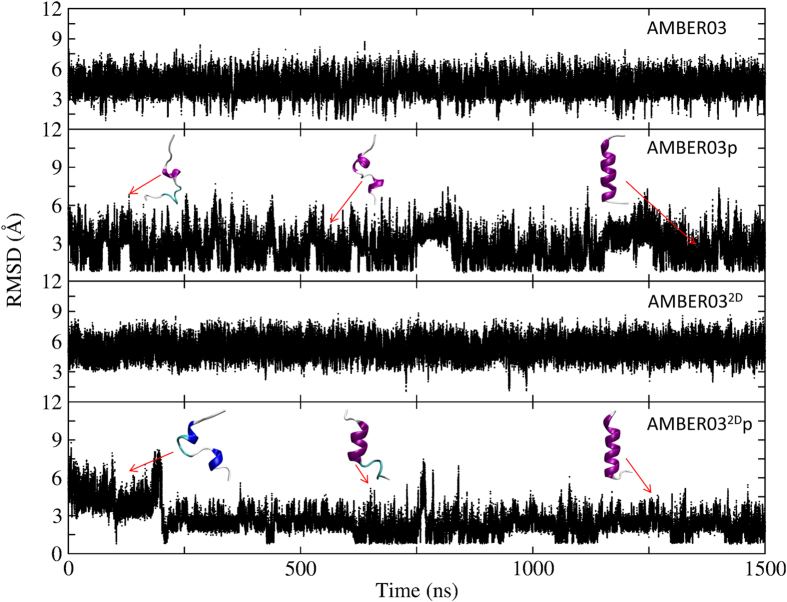
Time evolution of the backbone RMSD from the native structure at 300 K for 2I9 M, calculated from direct molecular dynamics simulations at room temperature using the AMBER03, AMBER03p, AMBER03^2D^, and AMBER03^2D^p force fields, respectively.

Direct molecular dynamics simulations that do not explore the entire phase space are also performed for 2I9M and trpzip2 to verify the results of REMD simulations and elucidate the folding mechanism. For 2I9M, the result is depicted in Fig. 8. Starting from a linear structure, the peptide does not fold to its native structure when using the AMBER03 or AMBER03^2D^ force fields. The average RMSDs are approximately 4.3 Å and 5.3 Å, respectively. When the polarization effect is incorporated, the peptide successfully folds to its native state with an RMSD distribution that peaks at 1.6 Å and 1.2 Å, respectively, for AMBER03p and AMBER03^2D^p force fields. Moreover, the peptide under the AMBER03p force field reaches a folded conformation earlier than under the AMBER03^2D^p force field, which is as expected due to the reduced helical propensity of this two-dimensional backbone potential. The representative structures under the AMBER03^2D^p force field reveal that the nucleation of the helix occurs near the termini and gradually extends toward the central residues. Finally, the peptide reaches its native state.

**Figure 9:**
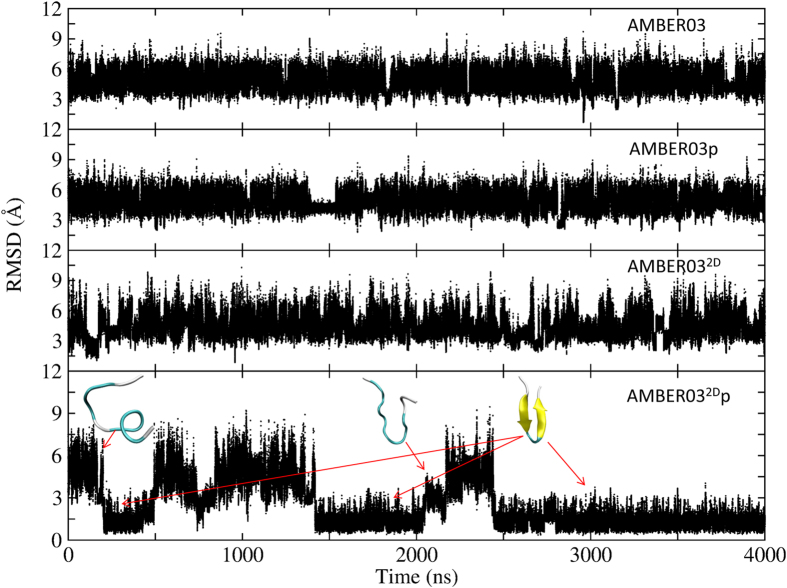
Time evolution of the backbone RMSD from the native structure at 300 K for trpzip2, calculated from direct molecular dynamics simulations at room temperature using the AMBER03, AMBER03p, AMBER03^2D^, and AMBER03^2D^p force fields, respectively.

The peptide trpzip2 also cannot fold into its native structure under AMBER03, AMBER03p, and AMBER03^2D^ force fields, with an average RMSD of 4.87, 4.91, and 4.44 Å, respectively, as shown in Fig. 9. However, under the AMBER03^2D^p force field, the peptide reaches a folded state for the first time at approximately 200  ns with a backbone RMSD below 1.6 Å. Residing in the folded structure for approximately 300 ns, the peptide unfolds and the RMSD increases to approximately 8.0 Å. The folded structure appears again at 1.4 μs and remains there for 0.6 μs. After another 0.5 μs in the unfolded state, this peptide stays in its folded structure until the end of the simulation. The representative structures in the folding process show that the central residues adopt either bended structures or β-turn structures, which is the direct consequence of the secondary structure preference of the AMBER03^2D^ force field and the polarization effect. However, β strands are not very stable. This result is quite consistent with the REMD simulation result showing that the hairpin is the dominant but not unique structure adopted by this peptide. This result is also consistent with the REMD simulation result showing that the folded state is preferred under the AMBER03^2D^p force field. The movies for 2I9M and Trpzip2 folding from direct molecular dynamics simulations at room temperature using the AMBER032Dp force field are shown in the Supplementary Information.

## Conclusion

The power of molecular dynamics simulations of proteins is limited by the accuracy and reliability of the force fields used. An unbalanced secondary structure propensity leads to failure in protein folding simulations. Moreover, the lack of an explicit polarization effect in pairwise force fields impedes the occurrence of ordered secondary structures. Recently, we optimized the AMBER force fields by replacing the uncoupled main chain torsion energy with a set of two-dimensional Fourier expansions, of which the parameters are fitted to the potential energy map resulting from high level quantum mechanical calculations of an alanine dipeptide^39^. Other force field parameters are kept intact as in the original AMBER force field. We name these new force fields AMBER99SB^2D^ and AMBER03^2D^. Their performance in producing the secondary structure distribution of an alanine dipeptide has been investigated in a previous study. Although the accuracy of this force field for this model peptide is guaranteed, its application to other dipeptides and long peptide chains with ordered structures may not be straightforward. This is not surprising because main chain torsions are coupled to side chains, and the electrostatic polarization effect along main chain hydrogen bonds does not emerge during parameterization. Specific torsion parameters for each residue and the potential refitting of χ1 and χ2 torsions are necessary.

In this work, folding simulations were performed for two peptides adopting either an *α*-helix or a *β*-hairpin in their native structures, although only limited improvement was observed in residues other than ALA. The AMBER03 force field was used for comparison. Both replica exchange and direct molecular dynamics simulations were conducted, and their results are consistent. In REMD simulations, high temperature replicas can easily surmount the energy barrier and explore the phase space more efficiently than standard molecular dynamics at room temperature. The intrinsic helical propensity of the AMBER03^2D^ force field is very low. Under this force field, peptide 2I9M primarily adopts random structures. No nucleation event was detected for the majority of its trajectory. For trpzip2, the preference of AMBER03^2D^ for extended structures leads to a large population of bended structures. The accumulation of this conformation is essential for folding. However, further advancement to the native structure has not been observed. Therefore, nonadditive effects must play a critical role in the folding of ordered secondary structures. These nonadditive effects can be captured with the AMBER03^2D^p force field, in which the native structures of both peptides dominate, indicating that this force field is well balanced between helical and β conformations and that the electrostatic polarization effect is indispensable.

The AMBER03 force field is biased toward helical structures, but the folding simulation of a short helical peptide, 2I9M, using the AMBER03 force field still fails. With the polarization effect enabled, this peptide successfully folds into its native structures as the global minimum in the free energy landscape. Folding a peptide with β-strands poses a challenge to the AMBER03 force field due to its excessive helical tendency. Only disordered structures can be observed in a simulation of trpzip2-folding employing the AMBER03 force field. After enabling the polarization effect, structures with short helical fragments can be detected.

This study implies that both secondary structure propensity and electrostatic polarization play critical roles in determining the folding structure of a protein. The lack of either of these two effects during parameterization may cause inconsistencies in systems outside the training set. We adopt the tactic of fitting the secondary structure propensity according to high level quantum mechanical calculations of short model peptides and accounting for the non-pairwise effect in long peptide chains by using electrostatic polarization. The feasibility of this tactic and the reliability of this new force field have been justified in this study as well as in our previous work^39^. These studies pave the way for the future development of polarizable force fields.

### Simulation Details

For dipeptides, tripeptides, and alanine tetrapeptide, the starting conformations used were linear structures generated using the LEaP module in AmberTools. The initial structure was immersed in the center of a truncated octahedral box of TIP3P water molecules, and all of the peptide atoms were no less than 12 Å from the boundary of the water box. To remove bad contacts before the simulation, we ran 20,000 steps of steepest descent followed by 20,000 steps of conjugate gradient energy minimizations. The relaxed structure was heated to its target temperature in 100 ps, during which all atoms in the protein were restrained with a force constant of 10 kcal/mol · Å^2^. The target temperatures were chosen to match experimental conditions; dipeptides were held at 303 K, GXG tripeptides at 298 K, homotripeptides at 300 K, and alanine tetrapeptide at 300 K. All bonds with hydrogen atoms were fixed using the SHAKE algorithm^69^. The particle mesh Ewald method with a 10 Å cutoff in real space was used to calculate electrostatic interaction. A Langevin thermostat with a collision frequency of 1.0  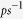 was used to regulate temperature^70^. Isotropic pressure coupling with a relaxation time of 2 ps was used to maintain the pressure to 1 atm. The integration time step was set to 2 fs. Trajectories were saved every 1 ps, and MD simulations were extended to 25 ns for each system (20 ns for dipeptides).

REMD simulations of 2I9M were performed with 12 replicas at 267.0, 283.0, 300.0, 328.0, 353.0, 380.0, 409.0, 439.0, 471.0, 506.0, 542.0, and 577.0 K. Those of trpzip2 were performed with the same number of replicas but at 255.00, 277.09, 300.63, 325.74, 352.50, 381.04, 411.51, 443.96, 478.65, 515.62, 555.04, and 597.08 K. All simulations started from a linear structure generated by the LEaP module in AmberTools. Each replica was heated to the target temperature in 100 ps after a local energy relaxation. The solvation effect was modeled using the generalized Born (GB) model developed by Mongan, Simmerling, McCammon, Case, and Onufriev^68^. This GB model is believed to have a local conformational propensity close to that of the TIP3P water model^67^. The salt concentration was set to 0.2 M, and the integration time step used was 1 fs. Surface area was computed using the LCPO model^71^. All bonds with hydrogen atoms were fixed using the SHAKE algorithm. Nonbonded interactions were fully counted without any truncations. Temperature was regulated using Langevin dynamics with the collision frequency of 1 ps^−1^. Swaps were attempted every 0.25 ps. After 2-ns of equilibration, simulations were extended to 160 ns per replica for 2I9M and 400 ns for trpzip2. Snapshots were saved every 0.25 ps. Direct simulations were performed at 300 K, and the simulation durations used were 1.5 and 4 μs for 2I9M and trpzip2, respectively. Other control options were chosen as in the REMD simulations. Clustering analysis was performed by the MMTSB^72^ package using the K-means clustering algorithm^73^. The MD simulations were performed using the AMBER11 Package with in-house modifications^74^.

Two reaction coordinates are defined in the study of trpzip2-folding. One is the collective hydrogen bond distance *d*, which is calculated by


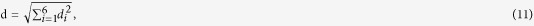


in which 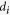 is the distance of the *i*th native hydrogen bond. This quantity is a metric of the formation of inter-strand hydrogen bonds. The other reaction coordinate is the stacking distance *l* defined as


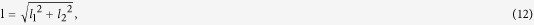


where 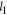 and 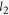 are the distance between TRP2 and TRP11 and that between TRP4 and TRP11, respectively. This coordinate is a metric of the stabilization force of π-stacking.

## Additional Information

**How to cite this article**: Gao, Y. *et al*. Correct folding of an α-helix and a β-hairpin using a polarized 2D torsional potential.*Sci. Rep.* **5**, 10359; doi: 10.1038/srep10359 (2015).

## Supplementary Material

Supplementary Information

Supplementary Video 1

Supplementary Video 1
