# Supplementary material for: Correct folding of an α-helix and a β-hairpin using a polarized 2D torsional potential
Source: Sci Rep. 2015 Jun 3;5:10359. doi: 10.1038/srep10359 (PMC5380191; doi:10.1038/srep10359)
Supplement: Supplementary Information [file srep10359-s1.doc]

**Correct folding of an α-helix and a β-hairpin using a polarized 2D torsional potential**

*Ya Gao^1^, Yongxiu Li^1^, Lirong Mou^2^, Bingbing Lin^1^, John Z. H. Zhang^1,3^*, *and Ye Mei^1,3,†^*

*^1^State Key Laboratory of Precision Spectroscopy, Department of Physics and Institute of Theoretical and Computational Science, East China Normal University, Shanghai 200062 China*

*^2^Institutes for Advanced Interdisciplinary Research, East China Normal University, Shanghai 200062 China*

*^3^NYU-ECNU Center for Computational Chemistry at NYU Shanghai, Shanghai China 200062*

†Author to whom correspondence should be addressed: [ymei@phy.ecnu.edu.cn](mailto:ymei@phy.ecnu.edu.cn)

**Video 2I9M-folding:** The movie for 2I9M folding from direct molecular dynamics simulations at room temperature using the AMBER03^2D^p force field.

**Video 1LE1-folding:** The movie for Trpzip2 folding from direct molecular dynamics simulations at room temperature using the AMBER03^2D^p force field.
